# Optimizing Spectral Utilization in Healthcare Internet of Things

**DOI:** 10.3390/s25030615

**Published:** 2025-01-21

**Authors:** Adeel Iqbal, Ali Nauman, Yazdan Ahmad Qadri, Sung Won Kim

**Affiliations:** School of Computer Science and Engineering, Yeungnam University, Gyeongsan 38541, Republic of Korea; adeeliqbal@yu.ac.kr (A.I.); anauman@ynu.ac.kr (A.N.); swon@yu.ac.kr (S.W.K.)

**Keywords:** eURLLC, 6G, healthcare Internet of things, artificial intelligence

## Abstract

The mainstream adoption of Internet of Things (IoT) devices for health and lifestyle tracking has revolutionized health monitoring systems. Sixth-generation (6G) cellular networks enable IoT healthcare services to reduce the pressures on already resource-constrained facilities, leveraging enhanced ultra-reliable low-latency communication (eURLLC) to make sure critical health data are transmitted with minimal delay. Any delay or information loss can result in serious consequences, making spectrum availability a crucial bottleneck. This study systematically identifies challenges in optimizing spectrum utilization in healthcare IoT (H-IoT) networks, focusing on issues such as dynamic spectrum allocation, interference management, and prioritization of critical medical devices. To address these challenges, the paper highlights emerging solutions, including artificial intelligence-based spectrum management, edge computing integration, and advanced network architectures such as massive multiple-input multiple-output (mMIMO) and terahertz (THz) communication. We identify gaps in the existing methodologies and provide potential research directions to enhance the efficiency and reliability of eURLLC in healthcare environments. These findings offer a roadmap for future advancements in H-IoT systems and form the basis of our recommendations, emphasizing the importance of tailored solutions for spectrum management in the 6G era.

## 1. Introduction

The recent proliferation of wearables has led to an exponential growth in lifestyle and health-tracking management systems. The delivery of healthcare and lifestyle improvement services has been transformed along with the manner of handling health emergencies [1]. Internet of Things (IoT) devices are pervasive within hospitals and other healthcare facilities, supporting several applications from continuous patient monitoring to tracking critical medical assets. IoT sensors have a compact form factor and significant accuracy, enabling remote real-time monitoring services. This enables clinicians to monitor the vital signs and identify potential health issues in patients before they reach a higher severity level, resulting in reduced fatality rates and medical costs. Besides that, such systems help optimize the operation of healthcare facilities by ensuring proper use of both material and human resources [2]. IoT facilitates seamless communication between healthcare facilities, caregivers, insurance providers, and patients. The health infrastructure should, therefore, be full of integration and responsiveness. A fast and reliable communication network is of critical importance to enable support for an increasing number and variety of healthcare IoT applications in a reliable, secure, and robust system [1,2].

The digital transformation is driven by ultra-reliable low-latency communication (URLLC), a requirement critical to the safety and effectiveness of modern medical treatments. In healthcare settings, even insignificant delays or interruptions in communication can have significant consequences. URLLC ensures the data from IoT devices are transmitted without extended delay and with the highest level of reliability, thereby maintaining the integrity of real-time patient monitoring and other mission-critical applications. This stringent demand for reliability and low latency is especially pertinent in scenarios such as telemedicine, remote surgery, and the management of life-support systems, where continuous, uninterrupted data flow is essential. As healthcare-IoT (H-IoT) ecosystems become more complex, with an increasing number of connected devices, the challenge of maintaining URLLC quality of service (QoS) amidst spectrum congestion and interference intensifies, making it a critical area of research and development efforts [3].

The massive deployment of IoT in healthcare has made healthcare more accessible. However, this democratization comes at a high cost of spectral utilization. Healthcare facilities are usually located in the city centers for easier accessibility. These areas are densely populated, which leads to overburdening the electromagnetic spectrum for wireless communication. For instance, urban hospitals have to deal with interference levels forcing data throughput down by 30% in peak hours of usage, with over 100 milliseconds of delay in transmitting data far above the 10-millisecond threshold necessary for applications that are critical, such as remote surgery [1,3,4]. This spectrum scarcity poses a challenge as an increasing IoT deployment requires more wireless channels to communicate efficiently. Therefore, severe competition for wireless spectrum can result in network congestion, which limits the performance of the overall healthcare system. Interference generated by dense IoT deployment is also among the leading causes of network performance degradation. These challenges create a need for highly efficient and robust spectrum management solutions that can not only cope with increasing spectrum demands but also minimize interference and improve the overall quality of the H-IoT system [2,5].

URLLC specifications form the fundamental requirements to ensure patients’ well-being. The time-sensitive nature of H-IoT networks entails the delivery of data within a tight delay tolerance, exceeding which could lead to fatal consequences. It is estimated that interference in urban hospital environments may cause packet loss rates as high as 15%, thereby degrading real-time health monitoring systems [3,4,6]. Communication reliability has the utmost significance as lost data can cause misdiagnosis or erroneous treatment. The challenges of limited spectrum availability and the high QoS requirements of URLLC underscore the critical need for innovative solutions that can manage these constraints effectively, ensuring that H-IoT systems operate in compliance with security and performance benchmarks [1]. The key performance indicators (KPIs) of healthcare systems in the fifth-generation cellular communication (5G) domain are analyzed in [7], where authors have reviewed experimental studies on achieving satisfactory performance in terms of these KPIs. The delay and reliability thresholds for H-IoT applications can be inferred from the cited case studies in [7], which are summarized in Table 1.

**Figure 1:**
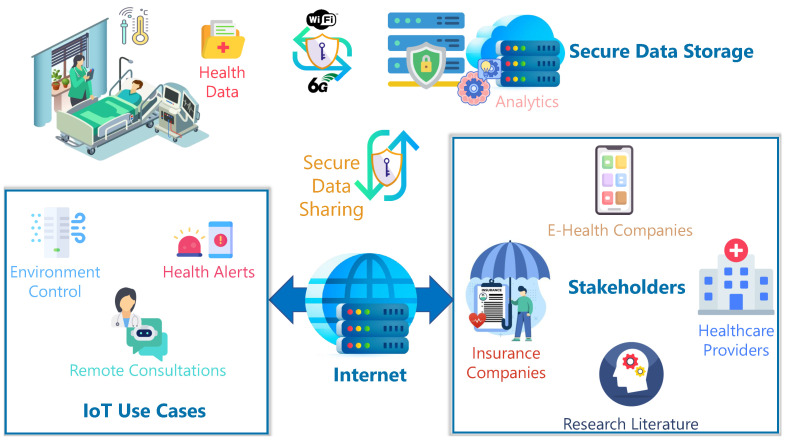
A general framework of H-IoT networks. The different use cases and stakeholders are identified.

Figure 1 illustrates the general architecture of H-IoT, elucidating the different faculties of an H-IoT system along with its use cases and stakeholders. Health data, generated from IoT devices, are securely forwarded to centralized systems for analysis, generating emergency alerts, and remote consultations. The stakeholders in this paradigm include healthcare providers, who can access real-time insights through secure data-sharing protocols that enable the best outcomes for patients. This is information flow in both directions, where healthcare providers or autonomous monitors can push feedback or counter-measures to IoT devices on environment control or personalized treatments.

### Contributions of This Work

The challenges in enabling sustained and reliable real-time communication for H-IoT are reviewed in this work. The contributions of this work can be summarized as follows.

- This work provides a clear understanding of an H-IoT system, its scope, and its underlying network operation, especially enhanced ultra-reliable low-latency communication (eURLLC). The focus of this review remains on sustained and reliable real-time communication for H-IoT, which acts as a background to understand critical challenges in efficient spectrum utilization.
- This work identifies the key challenges that limit the performance of real-time systems due to limitations in spectrum utilization.
- This work analyzes state-of-the-art strategies aimed at optimizing spectrum utilization in time-critical wireless networks, with a focus on the healthcare domain, including traditional and emerging approaches.
- This work compares emerging technologies and traditional approaches in terms of their performance gains. Artificial intelligence (AI)-based methodologies paired with advanced network architectures provide an insight into the emerging solutions and their potential to address the current challenges.
- The discussion on each of these solutions identifies research gaps and future research directions, focusing on experimental validation and practical deployment in real-world healthcare environments. Furthermore, this work presents some recommendations at the end based on the drawbacks of the existing solutions.

**Figure 2:**
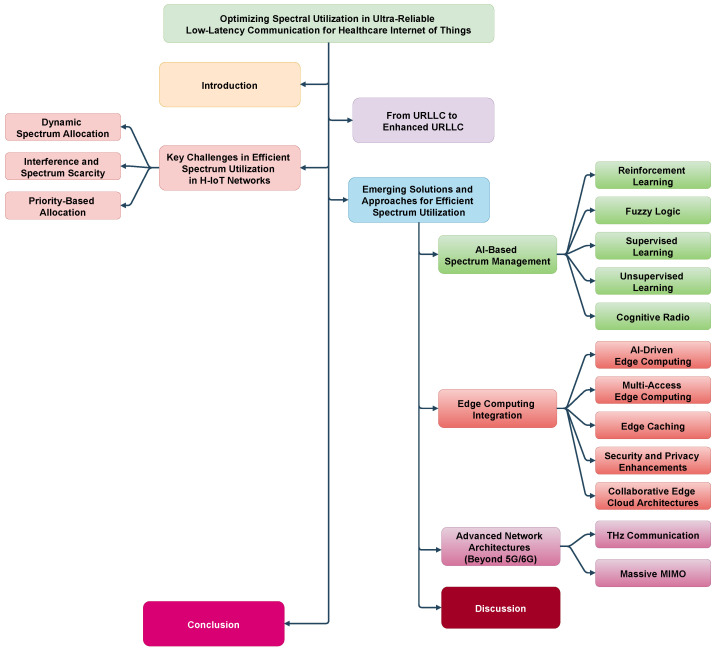
General structure of the study.

Figure 2 illustrates the structure of this survey. Section 1 highlights the significance of H-IoT and the key features of H-IoT. Section 2 introduces the underlying communication network features enabling H-IoT systems. Section 3 identifies the limitations of the current spectrum utilization methodologies for enabling eURLLC while Section 4 highlights the state-of-the-art solutions along with the future research directions based on the current solutions. Section 5 concludes the discussion.

## 2. From URLLC to Enhanced URLLC

URLLC is the defining feature of beyond 5G (B5G) networks and is intertwined in the design of sixth-generation (6G) cellular networks, designed to meet stringent requirements in delay and reliability performance [4]. The evolution of cellular communication specifications shifted the goalpost from maximizing the throughput and network capacity to enabling real-time communication with minuscule tolerance to transmission errors. The requirements of reliable communication in mission-critical applications including autonomous vehicles, smart industries, cobots, and healthcare drive this shift. The capabilities of URLLC will evolve further as 6G networks are rolled out commercially to support novel applications. The KPIs for 6G networks demonstrate the push for a further reduction of average end-to-end (E2E) latency to a sub-millisecond scale and reliability of 99.99999% [9]. These performance parameters define the eURLLC paradigm. The delivery of sustained performance at these levels addresses the increasing complexity and demands of emerging applications [10]. eURLLC networks are anticipated to include advanced technologies including terahertz (THz) communication, massive multiple-input multiple-output (mMIMO), and AI-driven network management. An interplay of all of these technologies is likely to play a critical role in securing real-time communications for future applications. Real-time remote surgery, telemedicine, and IoT-based monitoring applications can be potentially enabled by employing reliable, instantaneous, and secure communications.

The proliferation of H-IoT has sparked a rigorous research effort into the development of spectrum management and utilization strategies tailored to the demanding requirements of medical applications. Numerous research studies have explored the different aspects of spectrum allocation, such as efficiently managing the limited spectrum resources while ensuring the required reliability and low-latency communication necessary for critical healthcare applications. A comprehensive survey of technologies outlining the requirements and challenges of H-IoT networks is presented in [6]. The authors in [11] present a review of IoT applications in healthcare, focusing on the main application areas, components of IoT architecture, and enabling technologies. It highlights the challenges of security and privacy in IoT-based healthcare systems. The study in [12] investigates the use of cognitive radio (CR) for smart healthcare systems. It evaluates spectrum sensing using tree-based machine learning (ML) algorithms, aiming to improve the accuracy of spectrum utilization in healthcare settings. The authors in [13] discuss the integration of 5G and IoT in healthcare, emphasizing the potential for improved real-time communication, remote patient monitoring, and data management. It also addresses the benefits and challenges of combining these technologies. The systematic review in [14] analyzes the literature focused on IoT in healthcare, discussing enabling technologies, applications, and challenges. It provides insights into the current state of IoT in healthcare and identifies key areas for future research. The survey in [15] explores emerging IoT communication standards and technologies suitable for smart healthcare. It emphasizes low-power wireless technologies as key enablers for energy-efficient IoT-based healthcare systems and discusses major challenges in privacy and security. The study in [16] explores the transformative potential of the internet of medical things (IoMT) in healthcare. It discusses the integration of technologies like AI, ML, and blockchain into IoMT to improve patient care and health outcomes. The study also addresses the security and privacy concerns associated with IoMT devices and data. The study in [17] investigates the implications of IoT integration in the healthcare management domain. It presents a detailed discussion on how IoT can improve the functionalities of hospital management systems, focusing on the benefits and challenges of IoT adoption in healthcare settings. Table 2 summarizes the literature and highlights the key takeaways and research challenges with a focus on spectrum utilization.

Spectrum management strategies significantly affect the performance of H-IoT networks. Static resource allocation methods suffer from severe detriments when the number of users increases. This is due to the default allocation of a unique frequency band to specific applications. These methods often fail to adapt to the rapidly changing and unpredictable nature of the transmission features of electromagnetic signals in crowded environments filled with instruments causing interference. In dynamic environments, the bandwidth requirements fluctuate constantly due to varying device density and mobility. Moreover, the static allocation methods fail to satisfy all the needs of such a dynamic network; this approach fails to maximize throughput per Hertz per second. This rigidity usually leads to either under-utilization of the spectrum or congestion in certain bands, each compromising the real-time aspect of communication expected from the H-IoT network. The current spectrum management techniques can be improved to allocate resources based on priority while maintaining a reliable service for non-critical applications. Non-critical devices may occupy valuable spectrum resources at the expense of more crucial applications, exacerbating the challenges of maintaining eURLLC performance. These limitations underscore the need for more adaptive, intelligent, and context-aware spectrum management solutions that can respond in real time to the demands of H-IoT networks, ensuring that critical healthcare devices are always prioritized and supported with the required communication resources.

## 3. Key Challenges in Efficient Spectrum Utilization in H-IoT Networks

**Figure 3:**
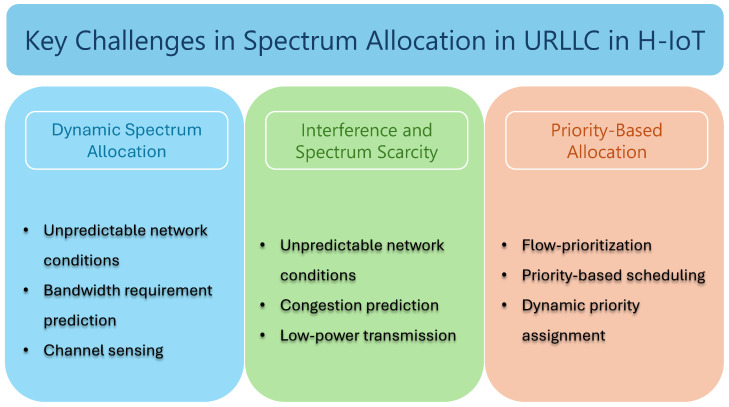
Key challenges in spectrum utilization for H-IoT networks.

As the number of H-IoT devices increases to serve diversified medical use cases, consolidated efforts are underway to realize reliable communication with a minimum delay and maximum reliability. Several challenges to achieving realistic eURLLC performance warrant attention, especially efficiently allocating the limited spectrum. This section delves into the key issues that need addressing to optimize spectrum utilization in H-IoT networks. This work explores the intricacies of dynamic spectrum allocation, which enables adapting to fluctuating network demands in real time, the pervasive problems of interference and spectrum scarcity that can hinder reliable communication, and the importance of priority-based allocation to ensure that critical medical devices receive the necessary bandwidth and low-latency support. Each of these challenges is crucial to maintaining the integrity and efficiency of healthcare IoT systems and demands innovative solutions to meet the stringent requirements of modern healthcare environments. Figure 3 summarizes the challenges in spectrum utilization in H-IoT networks.

### 3.1. Dynamic Spectrum Allocation

Recent studies have proposed solutions for dynamic spectrum allocation in H-IoT. For instance, one intelligent approach has been presented using fuzzy logic in the cognitive radio (CR) network to deal with inherent imprecision and ambiguity in spectrum sensing [18]. Another work proposed a deep multi-user reinforcement learning (RL)-based CR access strategy, which demonstrates enhanced wireless communication with improved user satisfaction [19]. Moreover, ML-based traffic prediction for 5G network dynamic spectrum allocation to reduce congestion and further improve network performance has also been explored [20]. An analytical model for dynamic spectrum sensing in CR incorporating blockchain-based management for more accuracy and security has also been proposed [21].

Despite these advancements, several challenges persist in implementing dynamic spectrum allocation in H-IoT. One of the primary challenges is predicting the demand for spectrum resources accurately [22]. The unpredictable nature of hospital environments makes it difficult to forecast bandwidth requirements, necessitating advanced algorithms and real-time monitoring systems to dynamically allocate spectrum resources based on current network conditions and anticipated demand. Another significant challenge is ensuring that critical devices are prioritized in the allocation process [23]. Failure to adequately prioritize these devices can lead to potential risks, including delayed data transmission or communication failures, which could have serious consequences for patient safety. Therefore, sophisticated prioritization mechanisms must be incorporated into dynamic spectrum allocation strategies to dynamically allocate resources based on the urgency and criticality of the communication.

### 3.2. Interference and Spectrum Scarcity

Interference and spectrum scarcity are among the major challenges in the optimization of spectrum allocation for H-IoT networks, particularly in dense urban populations. Many hospitals in urban cores usually face very high levels of electromagnetic interference due to highly dense deployments of IoT devices and medical equipment, along with coexisting radio access networks. It is shown that this interference can result in packet loss rates of up to 15% and a 30% reduction in data throughput during peak hours, which seriously impairs the reliability of time-critical applications such as remote monitoring and telemetry [3,6]. Furthermore, SINR levels in urban hospitals are often below the acceptable threshold of 10 dB, further degrading the quality of communication [3]. Spectrum scarcity further worsens these issues since the number of available wireless channels is reduced. Wearable health-monitoring devices are growing at an annual rate of 20%, further increasing pressure on the already crowded spectrum [24]. A case study involving a hospital utilizing multiple IoT-enabled devices for patient monitoring found that delays above 150 ms were experienced during peak operation times, which is far beyond the 10 ms tolerance needed for URLLC [1,4]. This underlines the importance of dynamic spectrum allocation techniques, enhanced interference management algorithms, and prioritization mechanisms toward ensuring reliable communications for the most critical medical devices.

Recent advancements to address interference and spectrum scarcity in H-IoT include novel solutions. One of the methods comprising intelligent dynamic spectrum access utilizes the concept of fuzzy logic in CR networks for handling imprecision and ambiguity in the estimation of spectrum sensing [15,18]. Other techniques involve the development of distributed RL algorithms for dynamic spectrum allocation in wireless communication to improve user satisfaction in CR-based IoT networks [19]. Other proposals explore ML-based traffic predictions to make better decisions on dynamic spectrum allocation in 5G networks, thereby reducing congestion and enhancing network performance [20]. An analytical model for dynamic spectrum sensing in CR has incorporated blockchain management to ensure high accuracy and security [21,25]. Emerging wireless sensor networks and IoT technologies can enable smart healthcare applications, with an emphasis on low-power wireless technologies for energy-efficient systems. These solutions collectively aim to improve spectrum utilization and mitigate interference for IoT networks that can be applied in H-IoT environments.

Hospitals are often located in urban areas with high levels of wireless traffic, leading to significant interference. This interference can adversely impact the reliability of the network required for critical healthcare applications. Interference can cause delays, data loss, and communication failures, which are unacceptable in healthcare settings where real-time data transmission is crucial. To mitigate these effects, several methods have been proposed. One approach is the implementation of advanced interference management techniques, such as interference cancellation and avoidance algorithms [26,27]. Furthermore, the use of CR technology allows for a dynamic spectrum access, enabling devices to switch to less congested frequency bands in real time [28]. Another method involves deploying small cells and heterogeneous networks to improve coverage and reduce interference. These strategies, combined with robust security measures, can enhance the reliability of eURLLC in H-IoT, ensuring that critical medical devices and applications function seamlessly even in high-interference environments.

### 3.3. Priority-Based Allocation

Prioritizing critical H-IoT devices over non-critical H-IoT devices during resource allocation is paramount. Recent works have proposed several solutions for prioritizing critical data transmissions. The authors in [29] proposed a new task scheduling and allocation approach called Prioritized Sorted Task-Based Allocation for healthcare monitoring implemented in IoT cloud-based architecture. The solution selects the best virtual machine for the execution of the health task, considering several factors that include wait time and expected time to process the task along with its criticality. The work in [30] proposes an intelligent patient health monitoring system (PHMS) based on optimized scheduling mechanisms using IoT-task orchestration architecture for monitoring the vital signs data of remote patients. It reduces task starvation and failure rates compared to conventional scheduling mechanisms. The authors in [23] discuss a priority-based resource allocation scheme along with smart channel assignment in a wireless body area network (WBAN) capable of energy harvesting. The proposed scheme prioritizes emergency and critical signals to avoid collisions, hence ensuring reliable data transmission. Authors in [31] present two hybrid approaches for resource allocation in WBANs based on the health data criticality. The first one considers joint AP association and channel allocation, while the second one includes a Stackelberg game with price updates to provide QoS for critical users. In [32], a prioritized scheduling (PS) scheme was proposed that outperforms the Earliest Deadline First (EDF) scheme for IoT-based healthcare applications. In the PS scheme, both the delay and size of data are considered for prioritizing critical healthcare tasks.

Ensuring critical medical devices are maintained before less critical ones is an important factor in H-IoT, mainly based on the aspect of patient safety. Life-support systems, real-time patient monitoring equipment, and emergency response tools must have seamless and reliable communication for proper performance in a clinical setting. By prioritizing these life-critical devices, they will obtain the bandwidth and resources required with no lag or malfunction, which might bring serious consequences to the patient’s health and safety. However, not many efficient prioritization mechanisms can act in real time through enactment. One of the challenges is that hospital environments are dynamic, being variable in demand for bandwidth and resources, hence making it difficult to maintain consistent prioritization. Given that this will require dynamic resource allocation proportional to urgency and criticality, advanced real-time monitoring systems are needed to support these algorithms. Moreover, prioritization mechanisms must ensure security and dependability to prevent risks like delays in transmission data or communication failures. The development of robust and adaptive prioritization strategies to respond to the ever-changing demands that healthcare environments impose is crucial to maintaining healthcare IoT system reliability and efficiency.

## 4. Emerging Solutions and Approaches for Efficient Spectrum Utilization

The rapid growth in H-IoT and subsequent high demand for eURLLC impose unparalleled pressure on spectral utilization, which mandates innovative and adaptive solutions. Novel approaches have to be devised since the traditional communication frameworks cannot support the high-level requirements concerning reliability, latency, and bandwidth unique to critical healthcare applications. These state-of-the-art developments in AI-driven spectrum management, advanced edge computing, and next-generation network architectures provide the necessary tools for further gains in spectral efficiency and also to meet high standards for H-IoT. The emerging approaches empower H-IoT systems to offer support for real-time patient monitoring, surgical assistance, and emergency response despite the complexity of challenges in serving the increasing number of users in the healthcare domain.

### 4.1. AI-Based Spectrum Management

AI and ML have emerged as powerful tools for predicting network conditions and dynamically optimizing spectrum allocation in H-IoT. These technologies enable real-time analysis and decision-making, which are crucial for maintaining the reliability and low-latency communication required in a medical setting. There have been several recent advancements in AI-based spectrum management solutions. The work [33] discusses how AI-collaborative IoT technologies can assist medical professionals with decision-making and develop a sustainable and smart healthcare system. The study highlights the potential of AI algorithms to enable machines to learn, make decisions, and process information more efficiently, thereby optimizing spectrum allocation. Authors in [13] highlights the potential for improved real-time communication, remote patient monitoring, and data management through the combination of 5G and IoT in healthcare settings. AI algorithms play a crucial role in optimizing spectrum allocation to support these applications. The efficiency of spectrum utilization can be mathematically expressed as follows:

(1) $$\eta =\frac{B\cdot \log_{2}\left(1+\mathrm{SINR}\right)}{W}$$

where *B* represents the bandwidth allocated to a device, SINR is the Signal-to-Interference-plus-Noise Ratio, and *W* is the total available spectrum bandwidth. This model quantifies spectrum efficiency and highlights how AI-based solutions optimize η by dynamically adapting allocation strategies in real time.

The work [34] focused on AI-driven dynamic spectrum allocation schemes that adapt to changing network conditions in healthcare environments. The AI algorithms prioritize critical medical devices to ensure low-latency and high-reliability communication. Authors in [35] explored the use of AI for intelligent spectrum management in future-generation communication systems, including 6G networks. The study discusses trending AI-based techniques, algorithms, and advanced models for spectrum management using cognitive radio and RL techniques. The survey in [18] reviews various intelligent decision-making techniques for dynamic spectrum access in CR networks, including fuzzy logic, RL, and game theory, and it discusses their applications in spectrum access and management. The AI models and techniques used in spectrum management in the literature can be categorized into the following categories.

#### 4.1.1. Reinforcement Learning (RL)

RL offers a promising paradigm to optimize the spectrum allocation problem in time-varying network scenarios and, hence, is highly suitable to the eURLLC scenario in H-IoT systems. Contrasting with traditional supervised learning, which learns with pre-defined labels, an RL model learns iteratively to obtain optimal policies through interactions with the environment directly, and it adapts to real-time feedback. This approach allows an agent to solve sequential decision problems with evaluative and delayed rewards. The latter is crucial for mitigation in complex fluctuating network conditions. RL algorithms, over time, learn and adaptively allocate the spectral resources across the time-varying network demands, thus enabling a fair and an efficient use of the spectrum. RL models can be computationally simple without requiring explicit training; they might be susceptible to unwanted delays when applied in time-sensitive applications. Balancing computational efficiency against adaptiveness in learning remains an open challenge in the effective deployment of RL [35,36].

Several works have been performed that compare different AI-based spectrum management techniques, demonstrating that RL algorithms achieve spectrum efficiency. In this respect, the work in [37] presents how the Vickrey–Clarke–Groves (VCG) auction-based algorithm outperforms the baseline allocation schemes in improving the spectrum utilization by 83%. The studies in [38,39] show that RL reduces latency, is simple to implement, and has less overhead. Therefore, they are very effective for urban hospital deployments. These results show the practical benefits of adopting AI-driven spectrum management techniques.

#### 4.1.2. Fuzzy Logic

Fuzzy logic systems are promising for the management of the spectrum in H-IoT systems. Real-time uncertainty and imprecision crop up within the communication environments. Noisy or incomplete data can, therefore, be interpreted and thereby effectively managed by a fuzzy logic system using linguistic variables and adaptive rule sets to ensure reliable spectrum allocation under dynamic networking conditions. While designing fuzzy rules is cumbersome and is usually considered a job for a domain expert, fuzzy logic is highly suitable in terms of interpretability and flexibility against ambiguities in signal conditions to support precise adaptive spectral management. Fuzzy logic, when combined with other AI techniques, may be viewed as one of the important enablers of robust and fast spectrum management frameworks to ensure the reliability of H-IoT systems operating under strict eURLLC requirements [18,40,41].

In further improving the management of the spectrum, a hybrid AI framework integrating RL with fuzzy logic can be employed. RL makes decisions dynamically by learning from network conditions, such as device density and interference levels, to reach an optimum allocation strategy. Fuzzy logic complements RL in managing uncertainties of demand and prioritization of critical devices. Fuzzy rules ensure that time-critical applications such as remote surgery will receive priority during spectrum allocation. It, thus, offers robust, adaptive management, which is appropriate for the heterogeneous nature of H-IoT systems.

#### 4.1.3. Supervised Learning

Supervised learning models, including neural networks, promise very good performance in predicting spectrum availability through pattern analysis from historical data, hence being useful in AI-based spectrum management in H-IoT systems. These models deliver a highly accurate performance with labeled datasets, enabling efficient spectrum allocation that can support real-time health monitoring and emergency interventions. Nevertheless, reliance on curated training data is considered to be challenging, since common healthcare settings generally have sparse, heterogeneous, and unpredictable data. Therefore, such a limitation may make these models hard to generalize for unknown conditions, rendering them less reliable in dynamic healthcare settings. While recently proposed approaches in the field of supervised learning, including Auto-ML, aim to improve model adaptability, there remains a need for more data-efficient and general methods that can handle real-world complexities in healthcare spectrum management [12,13,42,43,44].

#### 4.1.4. Unsupervised Learning

Unsupervised learning techniques, such as clustering, play a crucial role in the management of spectrum resources, especially in H-IoT. They can discover unseen patterns and outliers from large unlabeled datasets. A possible proposal is the K-means clustering that organizes healthcare devices and their communication needs into specific clusters, based on proximity, usage, or signal quality, to optimize network interactions. The clustering of similar devices can enable seamless communication with the devices within a cluster to communicate effectively with each other, enhancing bandwidth utilization and, thereby, reducing congestion in the network. Such mechanisms in H-IoT aid the real-time monitoring of patients. The clustering of health data may help in categorizing vital signs patterns across patient groups and prioritizing urgent ones. Furthermore, anomaly detection provides insight into unusual patterns in network traffic or device behavior that may occur from malfunctioning or an unexpected surge in data traffic; hence, this could allow for rapid troubleshooting and ensure system reliability. While these methods may not assign parts of the spectrum directly, their insights from data trends and anomaly patterns surely can feed into more refined and data-driven policies for spectrum allocation. Eventually, this will provide high levels of communication and reliability of healthcare systems, which increases the scalability and effectiveness of smart healthcare frameworks [35,45,46,47,48].

#### 4.1.5. Cognitive Radio

CR systems combined with AI capabilities play a very important role in the transformation of H-IoT through intelligent sensing of spectrum conditions and dynamic adaptation of transmission parameters. These resolve the issues of spectrum scarcity by facilitating the unlicensed users in opportunistically accessing underutilized spectrum bands, significantly improving spectrum efficiency without compromising the licensed primary users’ QoS requirements in communication. In particular, AI algorithms, especially ML and RL, advance the CR beyond just spectrum sensing, thus making the systems learn, adapt, and decide in real time; that is, from reactive to proactive spectrum management [49,50,51]. It can also dynamically allocate time, frequency, and spatial resources through AI, thus enabling an optimized network in the dynamic and usually unpredictable scenarios of healthcare. Deep learning (DL) can enable predicting spectrum usage patterns and, hence, move to a state to avoid interference with primary users, securing seamless, reliable connectivity that is essential for real-time patient monitoring and data transmission. However, robust sensing mechanisms are necessary as CR faces an environment of rapidly shifting network conditions. Further research effort is required on the enhancement of spectrum sensing accuracy, efficiency in the resource allocation process, and security in communications within AI-driven CNs, with efficient utilization achieving sustainable, responsive, and efficient healthcare systems [18,52,53,54].

Fundamentally, AI and ML use soft spectrum management to achieve network efficiency, spectrum allocation optimization, and low latency with dependable communication for H-IoT. Based on algorithms from clustering to RL and deep neural networks, these systems adapt to real-time status and change the network state to ensure sufficient spectrum availability alongside interference management across complex healthcare environments. AI-driven spectrum management is already envisioned to play a pivotal role in enabling eURLLC as healthcare systems continue to grow and demand increasingly robust communication networks. Latency in H-IoT networks can be quantified as follows:

(2) $$L=L_{\mathrm{trans}}+L_{\mathrm{proc}}+L_{\mathrm{queue}}$$

where $L_{\mathrm{trans}}$ is the transmission latency (inversely proportional to bandwidth), $L_{\mathrm{proc}}$ represents processing latency at the edge or cloud nodes, and $L_{\mathrm{queue}}$ denotes queuing delay due to network congestion. This relationship helps to identify bottlenecks and evaluate how edge computing solutions reduce *L* to meet stringent eURLLC requirements.

### 4.2. Edge Computing Integration

Edge computing plays a pivotal role in reducing latency by processing data closer to where they are generated, which is crucial for critical H-IoT systems. By allocating computational resources closer to the edge of the network, edge computing minimizes the physical distance of the data, thereby significantly reducing latency and improving response times. This is particularly important in modern healthcare environments where faster data processing can determine patient outcomes.

**Figure 4:**
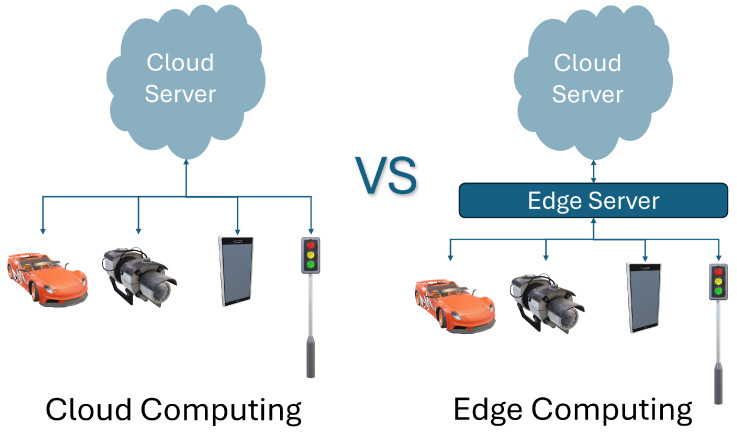
Functional difference between cloud and edge computing.

The traditional cloud computing model requires the data generated by IoT devices to be transmitted to remote centralized data centers for processing. The physical transmission adds delay due to the latency associated with the transmission characteristics of the medium. Edge computing implies that the sensed data are processed on or near the source of the data. Many applications require data analysis and counter-action closer to the end-user, especially in cases where the application requires a near-real-time response. Edge computing in the context of a hospital allows for the rapid processing of critical information such as vital signs in a patient, allowing for immediate detection of anomalies [55]. The difference between the data processing approaches in cloud and edge computing is illustrated in Figure 4.

The state-of-the-art techniques in the edge computing domain for enabling eURLLC, focusing on concepts such as AI-enabled edge computing, multi-access edge computing (MEC), edge caching, and security and privacy enhancements, are presented in this subsection. Collaborative edge–cloud architectures exploiting the advantages offered by edge and cloud resources to maximize performance and resource utilization are investigated. With growing IoT devices in healthcare, edge computing can provide an economical infrastructure that will scale up demand without overloading the centralized infrastructure. By distributing computational resources across edge nodes, healthcare institutions can handle the expanding range of connected devices and high volumes of data without compromising on performance or latency. This scalability is significant for hospitals while they adopt sophisticated IoT devices for patient monitoring, diagnostics, and real-time data gathering without degrading the quality of service and handling increased loads on the network [56]. Furthermore, it also leads to lower dependence on the cloud infrastructure, which, in turn, reduces the operational costs involved in maintaining the same for data transmission and storage [55,57]. Empirical studies demonstrate that the integration of edge computing with federated learning (FL) reduces latency by a factor of 11–38%, especially in applications requiring real-time data processing, such as remote patient monitoring. This approach provides benefits in preserving privacy by avoiding sharing the data for training. Simulation results further indicate that combining edge caching with blockchain increases the accuracy by 8–14% and amplifies data security [58].

#### 4.2.1. AI-Driven Edge Computing

The growth of AI and edge computing has transformed the decision-making processes in the eURLLC systems which are important to meet the stringent requirements of 5G and future 6G networks. Through edge-deployed AI algorithms, they can help interpret large datasets in real time, anticipate operational breakdowns, and allocate resources more efficiently, contributing to reliable and efficient operations [59,60,61]. This feature makes it vital for applications that need to have a latency of less than 1 ms and 99.99999% reliability in data transfer, like the smart grid and intelligent transport systems. The predictive analytics powered by AI can predict where network congestion is likely to occur and dynamically adjust the data flows within the network to ensure minimal latency and, therefore, overcome the limitations of conventional centralized architectures [62,63]. Moreover, the encapsulation of edge-centric artificial intelligence technologies like optimized ML models and low-latency communication protocols improves bandwidth efficiency as well as data security, enabling a smarter and more responsive healthcare framework. Through the combination of individual potentials of AI and edge, eURLLC can provide a platform for complicated services in diverse industries and create innovation and improvements in the real-time delivery of services [64,65].

#### 4.2.2. Multi-Access Edge Computing (MEC)

MEC is an enabling technology that makes it possible to bring cloud functionality closer to the user. It is possible for such systems to drastically reduce the time needed for cycling data back and forth, which is imperative in many use cases that require prompt action as in the case of remote monitoring of patients and emergency response systems [66,67,68]. MEC enhances mobile data and service exploitation by the processing of information and provision of useful services, within the RAN, simultaneously or in close succession with high-capacity new applications engineered for the 6G network. The confluence of MEC and 6G-enabling technologies not only significantly boosts the potential of eURLLC for use in healthcare but also opens many other possibilities such as autonomous vehicles, augmented and extended reality, and autonomous robotics. Given that the use of MEC in 5G networks is still in its nascent stages specific to deployment, further research and industry–academia collaboration must be fostered to fully exploit the existing resources as well as mitigate the issues of resource management, dynamic service placement, and security of edge computing environments [69,70,71].

#### 4.2.3. Edge Caching

Edge caching works by making a copy of the most frequently accessed data, patient records, and diagnostic images that are stored at network edge nodes, reducing dependence on servers far away. This enables significant reductions in latency, better reliability for access to time-critical data, and applications like robotic-assisted surgery and real-time patient monitoring. Advanced techniques, especially FL and deep reinforcement learning (DRL), further optimize this process. FL allows edge nodes to collaboratively cache content based on local usage patterns while guaranteeing patient data privacy, adapting models to localized needs, and reducing network load. Meanwhile, DRL-based systems predictably cache data through learning from network conditions and demands; thus, helping healthcare systems deal much more efficiently with fluctuations in the volume of data needs [55,72,73].

The integration of information-centric networking (ICN) with edge caching also presents a promising solution for healthcare applications that need real-time access to big volumes of data. ICN improves cache hit ratios and cuts delays in retrieval by giving priority to the content based on their type and frequency, thus supporting IoT-driven applications requiring massive volumes of data. These AI-driven caching solutions, thereby, create a strong foundation for compliance with the eURLLC in healthcare, enabling a wireless network that can resist the exacting demands of future digital health services with resilience, ultra-low latency, and optimization of resources according to the requirements [67,74,75].

Integration of edge caching with FL is considered one of the most promising directions for enhancing localized decision-making. Edge caching stores frequently accessed data locally, while FL trains models on local data independently at edge nodes without needing to centrally process the data. Integrating blockchain technology with edge computing will further enhance the security and transparency of spectrum management. Blockchain-based smart contracts can automate spectrum allocation based on the principles of fairness, hence preventing unauthorized use of the spectrum.

#### 4.2.4. Security and Privacy Enhancements

Edge computing aids in healthcare security and privacy where the sensitive data are processed locally while minimizing the utilization of central servers to reduce potential breaches. This can minimize the risk of data breaches and enhance privacy through localized approaches that enable healthcare providers to meet strict regulatory requirements, such as HIPAA and GDPR. Encryption with secure data transmission protocols is often performed at the edge to advance the safety of patient information while provisioning the low latency required for real-time communications in healthcare applications [66]. Furthermore, key techniques like advanced encryption and secure transmission protocols push to the edge, raising the difficulty level of data interception by malicious actors during transmission. This approach supports real-time processing, enhancing security while reducing latency in applications such as remote monitoring and diagnostics [55,76,77].

Federated Learning (FL) and Blockchain contribute to the added protection of patient data in healthcare, integrated into edge computing. FL enables the training of models locally on edge devices without raw data transmission, hence avoiding many privacy-related issues while being collaborative and secure at the same time. This is very relevant in H-IoT, which often has location-specific diverse data. Additionally, Blockchain provides an immutable and transparent ledger, strengthening authentication and access control at the edge. Taken together, these approaches can improve data privacy as well as guarantee sound security levels for healthcare applications in the emerging Internet of Everything (IoE) frameworks, which must support an increasingly decentralized and patient-centered healthcare ecosystem [78,79,80]. Edge computing increases reliability by distributing data processing operations on multiple nodes and reducing dependency on centralized servers, which have a single point of failure. This ensures that in mission-critical healthcare environments, critical data flows and processing are not interrupted in the case of network outages or high traffic. With IoT devices constantly monitoring patients and feeding vital information to medical staff, edge computing’s decentralized structure protects these processes from disruptions and offers a robust framework behind which data availability can consistently support high-quality patient care [55].

#### 4.2.5. Collaborative Edge–Cloud Architecture

The integration of edge and cloud computing in a hybrid architecture capitalizes on the unique strengths of each to create a responsive and efficient environment for eURLLC-enabled H-IoT networks. Accordingly, critical latency-sensitive data processing is performed at the edge, being positioned closer to the data sources for rapid response times with minimum delay. It moves the resource-intensive and not-so-time-critical tasks to the cloud, leveraging huge computation and storage resources in the cloud. This load-balancing approach further optimizes network performance and yields high reliability and low-latency computation-intensive healthcare applications [81,82].

Furthermore, this hybrid arrangement is enabled through resource allocation techniques, which distribute tasks, ensuring adherence to eURLLC requirements. It ensures mechanisms like joint power allocation, user association, and offloading strategies for the efficient use of both edge and cloud resources in healthcare applications where low latency is crucial for real-time monitoring and diagnostic applications. In summary, a hybrid architecture balances these computational loads across the network, enabling seamless data processing, enabling enhanced energy efficiency, and ensuring that critical medical information is always be presented to healthcare providers with minimum latency [83,84].

### 4.3. Advanced Network Architectures

B5G and 6G provide a significantly enhanced spectrum efficiency and support for eURLLC, which is quintessential for H-IoT applications. Advanced network architectures face the challenge of increasing demands for higher data rates, lower latency, and connectivity. The following section illustrates some of the potential technologies that are likely to change the design and deployment of future communication networks and their relationship with H-IoT. B5G and 6G technologies are expected to revolutionize H-IoT by providing unprecedented levels of connectivity and performance. THz communications and mMIMO are among the key technologies that will drive these enhanced capabilities [85]. mMIMO, in particular, facilitates the support of many devices with minimal interference, enhancing H-IoT by enabling reliable, continuous monitoring, and real-time data collection and analysis across numerous sensors and devices. Non-invasive imaging and diagnostics also become possible through THz communications. Unlike X-rays, THz waves do not ionize biological tissues, making them ideal for high-resolution, non-invasive imaging and diagnostic applications. This advancement allows for the early detection of health issues and a detailed assessment of patient conditions without invasive procedures, offering a safer and more accessible diagnostic alternative for patients [86].

#### 4.3.1. THz Communications

**Figure 5:**
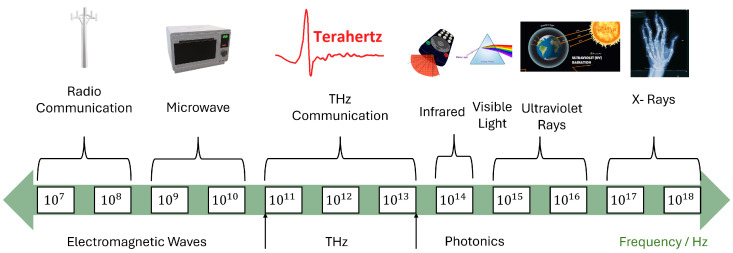
THz band in the electromagnetic spectrum.

THz communication works within the frequency range of 0.1-10 THz, opening a new dimension toward ultra-high data rates and huge bandwidths. Applications requiring ultra-high-resolution imaging and real-time data transfer are especially suitable and find wide applications in healthcare environments. Biological tissues can be penetrated by THz waves, thus enabling non-invasive imaging and diagnostics that have the potential to revolutionize branches of medicine dealing with oncology and cardiovascular health [87]. With the realization of B5G and 6G networks, THz technology is capable of meeting the increasing demand for URLLC a key to mission-critical applications. Furthermore, the integration of THz communications with edge computing will reduce energy consumption and enhance latency performance, making it a viable solution for real-time healthcare applications. Figure 5 places THz in perspective with the electromagnetic spectrum, also placing it in context as a key enabler of the emerging healthcare IoT landscape, underpinned by next-generation connectivity frameworks [86,88].

Though THz communication promises ultra-high data rates and bandwidth, it still faces some serious challenges impeding its practical deployment. Among these, the main challenge is to cope with the path loss, which limits the propagation distance of THz signals, due to the short wavelength and high susceptibility to scattering and reflection. Furthermore, molecular absorption, especially by water vapor in the atmosphere, seriously attenuates the THz signal, which further reduces the efficiency of the signal over long-distance transmission [89]. These call for novel solutions to be developed to make THz communication feasible. Advanced signal processing techniques, such as beamforming and Intelligent Reflecting Surfaces (IRSs), provide a potential solution that can redirect and amplify the THz signal by programmable metasurfaces, which overcomes path loss and extends the communication range effectively [90]. Interference management in dense deployments can be modeled as follows:

(3) $$\min I_{\mathrm{total}}=\sum_{i=1}^{N}\sum_{j=1,j\neq i}^{N}\frac{P_{i}\cdot P_{j}}{d_{ij}^{2}}$$

where $I_{\mathrm{total}}$ is the total interference, $P_{i}$ and $P_{j}$ are the transmit powers of devices *i* and *j*, and $d_{ij}$ is the distance between them. This model demonstrates how IRS can reduce interference by optimizing signal redirection and power levels. Moreover, the development of channel coding and channel modeling is crucial to reach an optimum solution in data transmission over the THz band. New encoding schemes such as polar code and low-density parity-check (LDPC) code will provide better error-correcting against molecular absorption. Moreover, these might be combined with accurate channel models, considering the peculiar characteristics of wave propagation, thus enabling the design of more robust and efficient tailored communication systems for deployment scenarios. These are innovations that drive practical implementation in THz communication for various emerging applications such as high-resolution imaging and healthcare IoT [10,91].

#### 4.3.2. Massive MIMO

mMIMO allows for a very large number of antennas to be used on the transmitter and the receiver, significantly improving the spectral efficiency, reliability, and connectivity. The capability of supporting an enormous number of devices in H-IoT, with robust and low-latency communication, is highly crucial to mission-critical medical applications such as remote patient monitoring and emergency diagnostics. mMIMO leverages channel hardening and favorable propagation properties—reducing interference and enhancing signal quality, even in dense device environments—to meet the stringent requirements of eURLLC [92,93]. The transceivers for mMIMO not only enhance reliability but also can support interference mitigation techniques, such as cell-free massive MIMO (CF-mMIMO) and grant-free access schemes. Special emphasis is put on CF-mMIMO, which can enable the eURLLC performance by spatially distributing the antennas across multiple access points. It provides better connectivity and resilience against interference, especially in dynamic healthcare environments. Therefore, it enables applications that require eURLLC, such as real-time telemedicine and remote surgery [94,95].

The large-scale deployment of an mMIMO approach for H-IoT faces several challenges caused mainly by high computational demand and power consumption. Similarly, with the computational complexity, there is a need to develop advanced channel estimation and beamforming algorithms. DRL in such resource management frameworks can, therefore, unlock the efficient use of resources for optimized sharing between slices of eMBB and eURLLC. DRL would prioritize uninterrupted connectivity of medical devices without compromising data rates or latency requirements for all other applications. Other challenges include pilot contamination, whereby pilots are shared across cells and induce interference, thus degrading the system’s performance. Such challenges have been approached using hybrid beamforming that reduces computational demand by turning to an analog–digital signal processing combination and energy-efficient hardware design such as a low-power amplifier and energy-harvesting antenna. Different pilot decontamination techniques, such as advanced pilot allocation schemes and machine learning-based channel estimation, can be used to mitigate pilot contamination. CF-mMIMO is another important concept that provides better coverage and interference mitigation in dense scenarios. These approaches enable massive MIMO systems to realize their potential for high spectral efficiency, reliability, and connectivity in next-generation communication networks. Hence, mMIMO acts as the cornerstone technology that has the potential to scale connectivity for H-IoT and, at the same time, provide a foundation for future-proof and high-performance communication networks in the healthcare vertical [85].

### 4.4. Discussion

The preceding subsections discuss several emergent solutions for efficient spectrum utilization in H-IoT systems, including AI-based spectrum management, integration of edge computing, and advanced network architectures such as THz communication and mMIMO. These solutions pave the way for more reliable, efficient, and scalable H-IoT networks, hence ensuring robust communication for critical healthcare applications. The solution to cope with real-time spectrum needs while prioritizing critical applications increasingly lies in AI-driven dynamic spectrum allocation. Among the advanced architectures, mMIMO, together with hybrid beamforming techniques, is prominent because these can improve spectral efficiency in densely populated environments. IRS and advanced channel coding techniques also hold immense promise for the unique challenges in THz communications. Future works will need to focus on experimental validation in real scenarios, possibly healthcare environments, and develop adaptive frameworks that can combine multiple techniques. Research could also be conducted on the decentralized approach of spectrum management using blockchain.

The summary of the various approaches to maximize the spectrum efficiency is tabulated in Table 3. This table provides an insight into the different underlying techniques and their advantages, drawbacks, and applicability to H-IoT systems.

## 5. Conclusions

The data transmission speed and reliability are crucial to patient safety and the efficiency of medical treatments over H-IoT networks. However, as the number of connected devices increases, ensuring spectrum availability and efficient utilization is essential to guarantee eURLLC performance. The significance of advanced spectrum management techniques in tackling challenges posed by healthcare settings, such as the need to prioritize essential medical devices and the dynamic nature of network demand, is highlighted in this work. Emerging solutions like dynamic spectrum management, AI-driven technologies, and next-generation network architectures offer intriguing possibilities to improve spectrum efficiency and satisfy the stringent demands of H-IoT applications, while traditional approaches, such as static spectrum allocation, frequently fall short in such circumstances. The fact that these solutions are still far from realizing their full potential suggests the necessity of continuing research and advancement in this field. Future research must concentrate on creating spectrum management strategies that are more intelligent and flexible, capable of instantly adapting to the changing demands of healthcare networks. Additionally, developing strong and dependable communication systems that can support the upcoming generation of H-IoT applications requires an interdisciplinary approach that combines knowledge from network technology, AI, and healthcare.

## Figures and Tables

**Table 1:** Upper limits on delay and reliability performance for healthcare applications. Monitoring applications have a higher delay threshold compared to haptic applications such as remote surgeries [8].

Application	Delay (ms)	Reliability (BER)
Healthcare Monitoring (Heart rate, stress levels, blood pressure)	250	$10^{-3}$
Remote Surgery	10	$10^{-4}$

**Table 2:** Summary of existing studies on enabling technologies and challenges for H-IoT networks.

Study	Focus Area	Methodology	Key Findings	Limitations
Qadri et al. [6]	Novel technologies enabling H-IoT	Comprehensive review	Survey of emerging technologies to power H-IoT	No empirical results presented
Ahmadi et al. [11]	IoT applications in healthcare	Comprehensive review	Identified main application areas, components of IoT architecture, and key technologies Highlighted security and privacy challenges	Limited to a review and lacks empirical data
Raza et al. [12]	Cognitive radio for smart healthcare	Spectrum sensing using tree-based ML algorithms	Improved accuracy of spectrum utilization in healthcare settings	Focused on a specific algorithm, may not generalize to all settings
Butt at al. [13]	Integration of 5G and IoT in healthcare	Discussion and analysis	Emphasizes potential for improved real-time communication, remote patient monitoring, and data management. Addresses benefits and challenges	Theoretical discussion; lacks practical implementation
Mohamad et al. [14]	Enabling technologies and applications of IoT in healthcare	Systematic review	Provides insights into the current state of IoT in healthcare. Identifies key areas for future research	Review-based, lacks experimental validation
Gardavšević et al. [15]	Emerging IoT communication standards for smart healthcare	Review paper	Emphasizes low-power wireless technologies as key enablers for energy-efficient IoT-based healthcare systems. Discusses privacy and security challenges	Survey-based, may not cover all emerging technologies
El et al. [16]	Internet of Medical Things (IoMT) in healthcare	Exploration and analysis	Discusses integration of AI, ML, and blockchain into IoMT; addresses security and privacy concerns	Exploratory; lacks empirical evidence
Almotairi et al. [17]	IoT integration in healthcare management	Detailed discussion	Presents benefits and challenges of IoT adoption in healthcare settings. Improves functionalities of hospital management systems	Discussion-based; lacks a practical implementation

**Table 3:** Comparison of emerging solutions for spectrum utilization.

Solution	Core Technology	Advantages	Challenges	Suitability for Healthcare IoT
RL	AI-based solution	Learns optimal policies through interaction with the environment [35]. Adapts to changing network conditions [38]. Achieves twice the throughput performance compared to slotted ALOHA) [96].	Requires significant training during exploration [35].	Low complexity allows for implementation in resource-constrained devices. Suitable for wireless networks.
Fuzzy Logic	Many-Valued Logic Approach	Handles uncertainty and imprecision. Useful in environments with incomplete or noisy data. (Achieves a maximum of 11 Gbps throughput for V2X [97].	Designing fuzzy rules can be complex [98]. Requires expert knowledge [99].	It does not require a high-specification processing device.
Supervised Learning	AI-based solution	Predicts spectrum availability based on historical data. Achieves a 60 Mbps data rate, which is almost twice that of a random search [95]. Effective with well-labeled datasets. A 12% enhancement in the prediction accuracy [100].	Struggles with generalization in unseen conditions [13]. Dependent on high-quality labeled data [101].	Can predict anomalies proactively to avoid delays.
Unsupervised Learning	AI-based approach	Identifies patterns and outliers without labeled training data. A 78% improvement in average packet arrival rate [102]. Useful for exploratory data analysis. A 12.7% improvement in energy efficiency [103].	May not provide precise control over spectrum allocation [35].	Suitable for efficient pattern recognition. Suitable for predicting issues.
Cognitive Radio	Utilizes unused spectrum	Enhances spectrum efficiency by allowing secondary users to access underutilized bands [52]. Dynamically adjusts transmission parameters. A 28% increase in spectrum efficiency [104,105].	Requires robust sensing mechanisms [28,106]. Potential for interference with primary users.	Effective in environments with variable spectrum availability.
Edge Computing	Distributed Computing	Achieves a maximum of 40% reduced latency [107]. Enhances data processing at the edge [108].	Requires robust infrastructure [109]. Security and privacy concerns [110].	Suitable for real-time data processing and analysis. Effective for applications requiring a low latency.
Terahertz Communications	THz Frequency Bands	High data rates and bandwidth. Achieves 200 Gbps at 100 GHz using QPSK [111]. Non-invasive imaging and diagnostics are used for blood cell detection, cancer cell characterization, bacterial identification, and biological tissue discrimination [112].	High atmospheric absorption [113]. Limited range [86].	Suitable for high-resolution imaging and real-time data transmission. Effective for non-invasive diagnostics.
Massive MIMO	Large Antenna Arrays	Supports a large number of devices [51]. Improves spectral efficiency and reliability; 38 bits/s/Hz using 500 antennas [114].	High computational complexity [115]. High power consumption [85].	Ideal for environments with many connected devices. Ensures reliable and low-latency communication.
